# Effects of protein intake and gender on body composition changes: a randomized clinical weight loss trial

**DOI:** 10.1186/1743-7075-9-55

**Published:** 2012-06-12

**Authors:** Ellen M Evans, Mina C Mojtahedi, Matthew P Thorpe, Rudy J Valentine, Penny M Kris-Etherton, Donald K Layman

**Affiliations:** 1 Department of Kinesiology, University of Georgia, 101A Ramsey 300 River Road, Athens, GA, 30602, USA; 2 Division of Nutritional Sciences, University of Illinois at Urbana-Champaign, 905 S Goodwin Ave, Urbana, IL, 61801, USA; 3 Department of Kinesiology and Community Health, University of Illinois at Urbana-Champaign, 906 S Goodwin Ave, Urbana, IL, 61801, USA; 4 Department of Nutritional Sciences, The Pennsylvania State University, 301 Chandlee Laboratory, University Park, PA, 16802, USA; 5 Department of Food Science and Human Nutrition, University of Illinois at Urbana-Champaign, 905 S Goodwin Ave, Urbana, IL, 61801, USA

**Keywords:** Protein, Weight loss, Body composition, Gender

## Abstract

****Abstract**:**

Limited data on sex differences in body composition changes in response to higher protein diets (PRO) compared to higher carbohydrate diets (CARB) suggest that a PRO diet helps preserve lean mass (LM) in women more so than in men.

****Objective**:**

To compare male and female body composition responses to weight loss diets differing in macronutrient content.

****Design**:**

Twelve month randomized clinical trial with 4mo of weight loss and 8mo weight maintenance.

****Subjects**:**

Overweight (N = 130; 58 male (M), 72 female (F); BMI = 32.5 ± 0.5 kg/m2) middle-aged subjects were randomized to energy-restricted (deficit ~500 kcal/d) diets providing protein at 1.6 g.kg-1.d-1 (PRO) or 0.8 g.kg-1.d-1 (CARB). LM and fat mass (FM) were measured using dual X-ray absorptiometry. Body composition outcomes were tested in a repeated measures ANOVA controlling for sex, diet, time and their two- and three-way interactions at 0, 4, 8 and 12mo.

****Results**:**

When expressed as percent change from baseline, males and females lost similar amounts of weight at 12mo (M:-11.2 ± 7.1 %, F:-9.9 ± 6.0 %), as did diet groups (PRO:-10.7 ± 6.8 %, CARB:-10.1 ± 6.2 %), with no interaction of gender and diet. A similar pattern emerged for fat mass and lean mass, however percent body fat was significantly influenced by both gender (M:-18.0 ± 12.8 %, F:-7.3 ± 8.1 %, p < 0.05) and diet (PRO:-14.3 ± 11.8 %, CARB:-9.3 ± 11.1 %, p < 0.05), with no gender-diet interaction. Compared to women, men carried an extra 7.0 ± 0.9 % of their total body fat in the trunk (P < 0.01) at baseline, and reduced trunk fat during weight loss more than women (M:-3.0 ± 0.5 %, F:-1.8 ± 0.3 %, p < 0.05). Conversely, women carried 7.2 ± 0.9 % more total body fat in the legs, but loss of total body fat in legs was similar in men and women.

****Conclusion**:**

PRO was more effective in reducing percent body fat vs. CARB over 12mo weight loss and maintenance. Men lost percent total body fat and trunk fat more effectively than women. No interactive effects of protein intake and gender are evident.

## **Introduction**

The prevalence of overweight and obesity as a public health issue is well established in adult men and women; however, the distribution and storage of adipose tissue as well as the metabolic consequences of elevated levels of adiposity appear to be impacted by sex status. It is well established that females store greater amounts of adipose tissue compared to males, whether expressed as absolute amounts or in relation to body weight (i.e. percent body fat or %Fat) [1]. Furthermore, with regard to fat depot distribution, there is a dimorphic patterning characterized by the typical woman storing greater amounts in the lower body, particularly the gluteofemoral region, and the typical man having the abdomen as the favored depot [1]. Because of the influence of sex hormones on this depot, the majority of females assume a more androidal fat pattern after the menopausal transition [2]. With regard to central adiposity, comparing subcutaneous and visceral depots, women are more likely to have a larger subcutaneous depot whereas men store more visceral adipose tissue [3,4], although again, this preferential depot may be altered with reductions in estrogen with the postmenopausal transition [2].

It is well established that weight loss in overweight or obese individuals reduces risk for metabolic diseases associated with adiposity [5]. However, body composition changes that occur with weight loss typically include loss of both fat mass (FM) and lean mass (LM). For example, a weight loss of 10 % body mass with dietary energy restriction decreased both FM and skeletal muscle in obese women [6]. Research, although limited, suggests that there are sex differences in body composition changes resulting from weight loss. Men appear to lose both FM and LM at a similar rate, whereas women have been found to lose more FM than LM, with these changes being related to baseline body composition [7,8]. Furthermore, regionally, it appears that although both men and women lose FM from the abdominal area and the femoral region, men may lose more abdominal fat, and women may lose more femoral FM [7,9].

The National Heart, Lung and Blood Institute guidelines call for weight loss regimens that attenuate loss of LM while losing FM [5]. The most effective macronutrient composition of the diet for optimal body composition change during weight loss is currently a high priority research area [10] and may have implications for optimal body composition changes, especially in mid-life and older adults [11]. Our own research suggests that weight loss diets higher in protein and lower in carbohydrate (PRO) appear to promote marginally more weight loss, similarly to previous findings [12,13], but augment FM loss with a relative preservation of LM compared to a conventional higher carbohydrate/lower protein diet (CARB) [14] although not all studies are in agreement [15,16].

The dietary protein requirement is frequently expressed as 15-20 % of total energy [17]. This format may be inappropriate when energy intake is determined without respect to the absolute protein requirement. For example, 15 % energy from protein may fall short of the absolute protein requirement of 0.8 g·kg^-1^·d^-1^ during energy restriction, as in a weight loss diet. Theoretically, individuals with more LM, including males relative to females, may be particularly susceptible to suboptimal protein intakes during energy restriction, promoting a loss of lean body mass [17]. However, one study on body composition changes in response to PRO compared to CARB weight loss diets suggest that a PRO diet helps preserve LM in women more so than in men [9]. Insufficient data directly comparing male and female body composition responses to weight loss diets differing in macronutrient content are currently available to draw conclusions or develop dietary recommendations.

In this context, the purpose of this analysis was to examine sex differences in whole body and regional body composition changes, including FM and LM, resulting from weight loss in response to isocaloric PRO and CARB weight loss diets. We anticipated that the PRO diet would more favorably affect changes in body composition (i.e. promote FM loss and attenuate LM loss) in men, who have greater LM and may thus require more protein, than in women, compared to the CARB diet.

## **Subjects and methods**

### Overview

This study was a 12 mo two-center weight loss trial (4 mo active weight loss; 8 mo weight maintenance) using a parallel-arm randomized design. This gender comparison is a secondary analysis of the weight loss trial previously reported elsewhere [18,19]. Subjects were blocked by body mass index (BMI), sex and age then randomized into diets prescribing either a low carbohydrate to protein ratio (PRO group: ~ 1.6 g·kg^-1^·d^-1^ of protein or 30 % of energy from protein, 40 % carbohydrate and 30 % fat) or a high carbohydrate to protein ratio (CARB group: 0.8 g·kg^-1^·d^-1^ of protein or 15 % protein, 55 % carbohydrate and 30 % fat). Due to study design of the diet treatment, it was not possible to blind subjects and research staff to group assignment. (See *Diet Treatments* below.)

### Subjects

One hundred thirty men (n =58) and women (n =72) aged 40 to 56 y were recruited to participate in the weight loss study. Exclusion criteria were BMI < 26 kg/m^2^, body weight > 140 kg (due to DXA scanning bed constraints), smoking, any existing medical conditions requiring medications that impact primary or secondary outcomes of the study, i.e. use of oral steroids or use of anti-depression medication. This study was approved by the Institutional Review Boards at the University of Illinois at Urbana-Champaign and The Pennsylvania State University. Subjects provided written informed consent prior to participation in the study.

All subjects participated in a baseline evaluation period that included a 24-h food recall, instructions for weighing and recording of foods, two 3-d weighed food records during separate weeks and measurements of height and weight. This evaluation period was 10 to 20 d and served as an initial control period for each subject. During the baseline period, subjects were instructed to maintain stable body weight and to consume a diet similar to the past 6 mo. After the baseline period, subjects reported to the laboratory after a 12 h overnight fast for measurements of body weight and body composition.

### Diet treatments

The PRO diet prescribed dietary protein at 1.6 g^**.**^ kg ^-1**.**^d^-1^ (~30 % of energy intake) with a carbohydrate/protein ratio <1.5 and dietary lipids ~30 % energy intake. The CARB diet provided dietary protein equal to 0.8 g^**.**^kg^-1**.**^d^-1^ (~15 % of energy intake) with a carbohydrate/protein ratio > 3.5 and total fat ~ 30 % of energy intake. These diets were designed to fall within the Acceptable Macronutrient Distribution Range of the DRIs as established by the Institute of Medicine [20] with minimum RDA intakes for carbohydrates >130 g/d and protein >0.8 g^**.**^kg^-1**.**^d^-1^ and with upper ranges for carbohydrates <65 % and protein <35 % of total energy intake. The two diets were formulated to be equal in energy (7.10 MJ d^-1^, 1700 kcal d^-1^ for females; 7.94 MJ d^-1^, 1900 kcal d^-1^ for males), total fat intake (30 % of energy) and fiber (17 g/1000 kcal). Each diet group received a menu plan with meals for each day meeting established nutritional requirements [20] and dietary fat guidelines [21]. Diet differences between groups were designed to reflect direct substitution of foods in the protein groups (meats, dairy, eggs and nuts) for foods with high carbohydrate content (breads, rice, cereals, pasta and potatoes). The education guidelines for the CARB group followed the USDA Food Guide Pyramid [22] and emphasized restricting dietary fat and cholesterol with use of whole grain breads, rice, cereals and pasta. For the PRO group, the education guidelines emphasized use of high quality low fat proteins including lean meat, reduced fat dairy and eggs or egg substitutes. Both diets included 5 vegetable servings/d and 2 to 3 fruit servings/d.

### Education Program and Monitoring

Subjects were provided electronic food scales and instructed to weigh all food servings at all meals. Subjects were required to report two 3-d weighed food records during the baseline period prior to assignment to diet groups. Nutrient intakes were evaluated as mean daily intakes from the 3-d weighed records using Nutritionist Pro software (First DataBank Inc. 2003, San Bruno, CA). After baseline data collection, subjects received specific diet program instructions from a research dietitian including the menus, food substitutions and portion sizes. Throughout the 12-mo study, subjects were required to attend a 1 h meeting each week at the weight management research facility. Meetings were specific for each treatment group and directed by research dietitians who provided diet and exercise information, answered questions and reviewed diet records for treatment compliance. Each week, subjects were weighed in light clothing without shoes and turned in 3-d weighed food records.

The education program focused on diet compliance with some exercise guidance. Activity guidelines emphasized physical activity lifestyle recommendations based on the NIH Guidelines for Weight Management [5]. These guidelines recommend a minimum of 30 min of walking 5 d/wk. Participation in physical activity for the groups was voluntary. Physical activity was monitored using daily activity logs and armband accelerometers (BodyMedia, Cincinnati, OH) worn 3 d/mo. Activity logs were collected each week. Based on these measurements, subjects averaged less than 100 min/wk of added exercise. These results were similar between diet treatment groups.

### Body composition

After 4, 8 and 12 mo, subjects reported to the laboratory after a 12 h overnight fast for measurements of body weight and body composition. Body weight was measured using an electronic scale (Tanita, Model BWB-627A, Tokyo Japan). Height was measured using a stadiometer. Body composition was determined by dual energy X-ray absorptometry (DXA; Illinois: Hologic QDR 4500A, software version 11.1:3; Penn State: Hologic QDR 4500 W, software version 12.5) and scans for a given individual were analyzed by the same technician. A regional analysis was performed per manufacturer guidelines, which involved placing lines bisecting the femoral neck and the glenohumeral joint. Appendicular lean mass was determined by summing lean soft tissue mass from leg and arm subregions. The appendicular skeletal muscle index was calculated as total appendicular lean mass / height² in meters.

### Data Analyses and Statistics

Data were screened for normality and outliers. Change in dietary intakes and body composition were compared across time, with respect to diets and sex using unstructured repeated measures ANOVA models. Models were applied to intakes of total energy, protein, carbohydrate and fat, as well as weight and weight loss from baseline, total body LM, FM and %Fat and regional %Fat of the legs and trunk. The ratio of FM of the trunk vs. the legs was also modeled, as well as the distribution of fat mass of the legs and trunk, that is, the proportion of total body FM contained in these regions. These parameters permitted contrasting of the primary site of weight loss across diets and sexes. All models used intent-to-treat analysis and tested for all two- and three-way interactions of sex, diet and time. Although statistical inference was rooted in the repeated measures models, change scores were calculated and are utilized for baseline-adjusted visual presentation of the data. All analyses were performed using SPSS version 14 (SPSS, Inc., Chicago, IL). Statistical significance was defined as α = 0.05. Reported values are means ± standard errors.

## **Results**

Eighteen of 36 CARB females had withdrawn from the study at 12 months, as well as 18 of 30 CARB males, 14 of 36 PRO females and 9 of 28 PRO males. Total body weight, FM and LM loss and dietary intakes have been reported previously [18,19]. Dietary intakes are reiterated briefly here, whereas body composition outcomes reported here focus on sex differences. Participants lost 8.2 % of baseline body weight (95 % CI, 7.5-8.9) at 4 mo and 10.5 % (8.9-12.0) at 12 mo, with no differences by diet, sex, or their interaction (all *P* > 0.2). By 4 mo, energy intake was aligned approximately at prescribed levels, and protein and carbohydrate intakes diverged according to diet as prescribed (diet x time interaction P < 0.05; Table 1). Diets were similar in energy intake across diets through the intervention, although they differed between PRO and CARB males at baseline (Table 1).

**Table 1:** **Energy and macronutrient intakes in adults on the CARB and PRO diets**^1^

		**Baseline**		**4 months**		**12 months**	
		CARB	PRO	CARB	PRO	CARB	PRO
Energy, MJ	F	8.73 ± 1.90	8.74 ± 1.47^a^	6.11 ± 1.29^c^	6.14 ± 1.06^ac^	6.23 ± 1.05^c^	6.48 ± 1.55^ac^
	M	8.55 ± 2.78^b^	11.4 ± 3.20	6.61 ± 2.05^c^	7.45 ± 1.74^c^	7.68 ± 1.88	8.03 ± 1.67^c^
Protein, g/d	F	78 ± 18	78 ± 17^a^	64 ± 11^bc^	103 ± 23^abc^	64 ± 14^b^	101 ± 23^abc^
	M	87 ± 28^b^	114 ± 43	75 ± 15^bc^	129 ± 26^bc^	76 ± 16^b^	134 ± 32^bc^
Protein, %E	F	15.0 ± 3.5	14.9 ± 3.3	17.5 ± 3.0	28.1 ± 6.3	17.2 ±3.8	26.1 ± 5.9
	M	17.0 ± 5.5	16.8 ± 6.3	19.0 ± 3.8	29.0 ± 5.8	16.6 ±3.5	28.0 ± 6.7
Carbohydrate, g/d	F	268 ± 67	266 ± 60^a^	208 ± 47^bc^	154 ± 36^c^	210 ± 48^bc^	154 ± 43^c^
	M	251 ± 82^b^	324 ± 118	244 ± 60^b^	172 ± 55^c^	267 ± 86^b^	185 ± 56^c^
Carbohydrate, %E	F	51.4 ± 12.9	51.0 ± 11.5	57.0 ± 12.9	42.0 ± 9.8	56.5 ± 12.9	39.8 ± 11.1
	M	49.2 ± 16.1	47.6 ±17.3	61.8 ± 15.2	38.7 ±12.4	58.2 ± 18.8	38.6 ±11.7
Fat, g/d	F	77 ± 22	79 ± 23^a^	44 ± 14^c^	52 ± 10^ac^	49 ± 17^c^	60 ± 19^ac^
	M	72 ± 30^b^	108 ± 40	42 ± 14^bc^	66 ± 17^c^	55 ± 18^bc^	74 ± 15^c^
Fat, %E	F	33.2 ± 9.5	34.1 ± 9.9	27.1 ± 8.6	31.9 ± 6.1	29.6 ± 10.3	34.9 ± 11.0
	M	31.7 ± 13.2	35.7 ± 13.2	23.9 ± 8.0	33.4 ± 3.8	27.0 ± 8.8	34.7 ± 7.0

^1^ Values are mean ± SD, n = 130. %E = percentage of energy. Compared using an unstructured repeated measures model, α = 0.05, with no adjustment for multiple comparisons. ^a^ Differs from males within time and diet. ^b^ Differs from PRO within time and gender. ^c^ Differs from baseline within diet and sex. CARB = higher carbohydrate diet. PRO = higher protein diet. 4 month = 4 months of weight loss intervention from baseline. 12 months = 4 months of weight loss and additional 8 months of weight maintenance from baseline.

Protein intake also approached prescribed levels in the PRO (1.37 ± 0.04 g kg^-1^·d^-1^ or 29 ± 0.6 percent of energy) and CARB (0.82 ± 0.03 g kg^-1^ ·d^-1^ or 18 ± 0.3 percent of energy) groups at 4 mo. Protein intake was similar at 4 and 12 mo (Table 1). Fat intake increased slightly from 4 to 12 mo irrespective of diet (*P* < 0.05). Carbohydrate intake declined in PRO participants to accommodate additional protein, as expected, though fat intake was mildly elevated in PRO vs. CARB participants (Table 1). Intakes of all nutrients were generally higher in men (Table 1). Physical activity was similar across diet and sex and timepoints (*P* > 0.10).

Although men lost more total weight than women (*P* = 0.04 for sex x time interaction), this effect was eliminated when weight loss was expressed as a percentage of baseline weight (Table 2). Significant interactions for both diet x time (*P* = 0.03) and sex x time (*P* < 0.01) were observed for loss of whole body FM, although diet effects within each time point were not significant (Table 1). A similar pattern emerged for LM (*P* = 0.03 for diet x time; *P* < 0.01 for sex x time). The three-way interaction of diet x sex x time was not significant at any site for LM or FM.

**Table 2:** **Body composition in males (M) and females (F) on the CARB and PRO diets**^1^

		**Baseline**		**4 months**		**12 months**	
		CARB n = 66	PRO n = 64	CARB n = 51	PRO n = 52	CARB n = 30	PRO n = 41
Weight, kg^2^	F	87.6 ± 11.4^a^	85.1 ± 12.0^a^	81.4 ± 12.3^ab^	78.4 ± 11.3^ab^	80.6 ± 12.9^ab^	77.6 ± 13.1^ab^
	M	100.1 ± 10.8	100.2 ± 16.4	89.7 ± 10.0^b^	92.3 ± 14.6^b^	85.9 ± 8.1^b^	90.2 ± 16.1^b^
Weight loss %^2^	F	-	-	−7.3 ± 3.8^b^	−8.3 ± 3.2^b^	−10.3 ± 6.1^b^	−9.5 ± 6.0^b^
	M	-	-	−8.5 ± 4.0^b^	−9.0 ± 3.4^b^	−9.8 ± 6.5^b^	−12.1 ± 7.6^b^
WB fat, kg^3^	F	36.8 ± 7.8^a^	34.6 ± 7.5^a^	33.0 ± 7.6^ab^	30.2 ± 6.5^ab^	32.8 ± 7.7^ab^	29.6 ± 7.9^ab^
	M	30.5 ± 5.5	28.7 ± 7.7	24.0 ± 4.6^b^	22.5 ± 6.8^b^	21.6 ± 4.6^b^	21.0 ± 7.3^b^
WB lean, kg^3^	F	48.9 ± 6.3^a^	48.3 ± 6.2^a^	46.9 ± 6.3^ab^	45.8 ± 6.8^ab^	46.3 ± 5.7^ab^	46.6 ± 6.9^ab^
	M	67.4 ± 8.1	68.6 ± 10.4	62.4 ± 7.6^b^	65.7 ± 9.3^b^	61.5 ± 5.1^b^	66.6 ± 10.8^b^
Appendicular lean mass, kg	F	21.0 ± 3.2^a^	20.9 ± 3.2^a^	20.0 ± 3.1^ab^	19.7 ± 3.3^ab^	18.9 ± 5.1^ab^	19.9 ± 3.4^ab^
	M	30.6 ± 4.7	31.7 ± 6.0	28.1 ± 4.0^b^	30.5 ± 5.5^b^	28.0 ± 3.2^b^	31.0 ± 6.1^b^
Appendicular lean mass / m²	F	7.7 ± 1.0^a^	7.9 ± 1.2^a^	7.3 ± 1.0^ab^	7.5 ± 1.2^ab^	6.9 ± 1.9^ab^	7.5 ± 1.1^ab^
	M	9.9 ± 1.4	10.0 ± 1.3	9.2 ± 1.3^b^	9.6 ± 1.3^b^	9.1 ± 1.0^b^	9.7 ± 1.3^b^

^1^ Values are mean ± SD. Compared using an unstructured repeated measures model, α = 0.05, with no adjustment for multiple comparisons. ^2^ Weight measured with a digital scale. ^3^ Measured with dual-energy X-ray absorptiometry (DXA). ^a^ Differs from males within time and diet. ^b^ Differs from baseline within diet and gender. CARB = higher carbohydrate diet. PRO = higher protein diet. 4 month = 4 months of weight loss intervention from baseline. 12 months = 4 months of weight loss and additional 8 months of weight maintenance from baseline.

**Figure 1:**
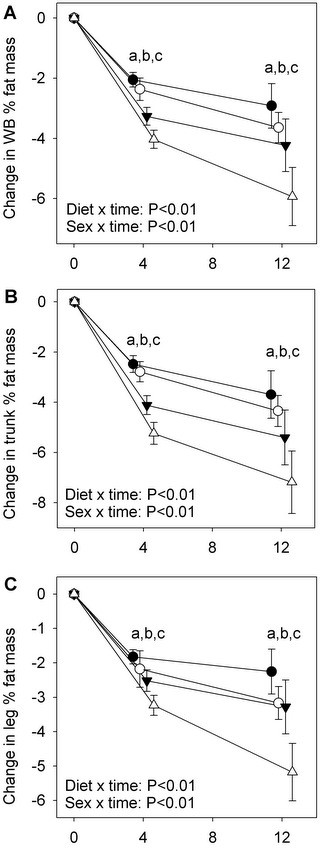
**Change in % fat mass of the whole body (WB), trunk and legs in adult men and women prescribed to a higher protein (PRO) or high carbohydrate (CARB) diet.** ∆: PRO males, ○: PRO females, ▼: CARB males, ●: CARB females. Values are mean ± SEM, n = 130, α = 0.05. Analysis performed using an unstructured linear mixed model including diet, sex, time and their two- and three-wayinteractions. No significant sex x diet x time interactions were observed. ^a^ significant effect of sex within time. ^b^ significant effect of diet within time. ^c^ all groups differ from respective baseline values.

No diet or diet x time interaction effect was observed on total body LM or appendicular LM. A decline in %Fat was found for women and men in both diets at the whole body, trunk and legs (Figure 1). Though %Fat improved for all subjects, improvements were more pronounced in men compared to women and in PRO compared to CARB participants (*P* < 0.01 for diet x time and sex x time effects; Figure 1). The three-way interaction of diet x sex x time was not significant at any site.

The share of total body fat stored at the trunk was also higher at baseline in men (mean difference 7.0 ± 0.87 %, *P* < 0.01), and experienced a greater decline in men (change of −3.0 ± 0.54 % at 12 mo) compared to women (change of −1.8 ± 0.32 % at 12 mo; *P* = 0.02 for sex x time interaction). As expected, the share of total fat carried in the legs was higher in females (mean difference at baseline 7.2 ± 0.85 %, *P* < 0.01), but the interaction of sex and time was not significant (*P* = 0.16). The effect of diet and its interactions with sex and time were not significant for either of these parameters.

**Figure 2:**
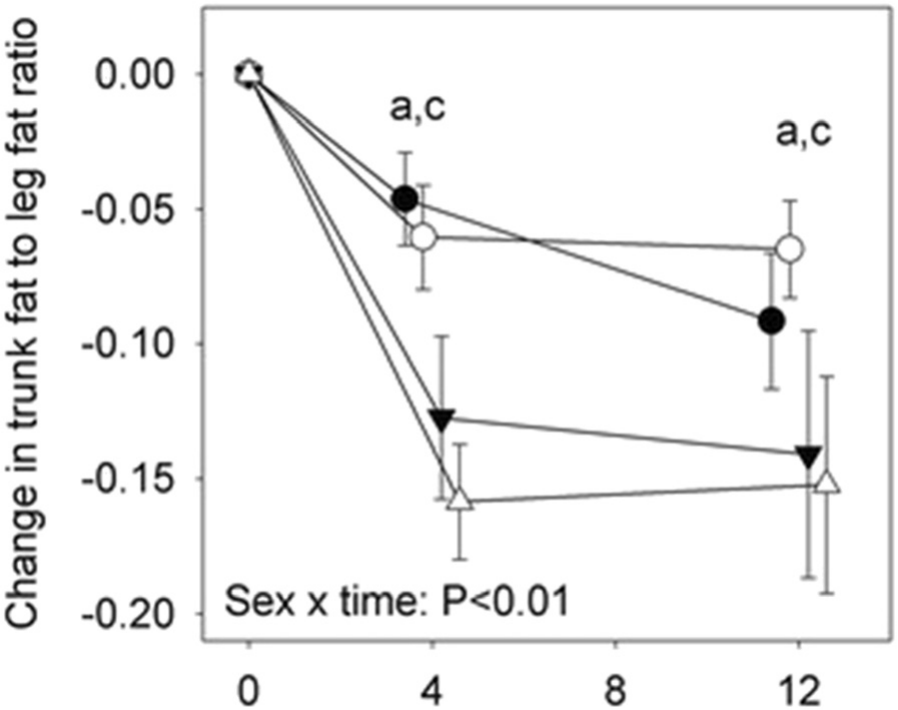
**Change in the ratio of trunk to leg fat mass for adult men and women prescribed a higher protein (PRO) or a high carbohydrate (CARB) diet.** ∆: PRO males, ○: PRO females, ▼: CARB males, ●: CARB females. Values are mean ± SEM, n = 130, α = 0.05. Analysis performed using an unstructured linear mixed model including diet, sex, time and their two- and three-way interactions. No significant sex x diet x time interactions were observed. ^a^ significant effect of sex within time. ^c^ all groups differ from respective baseline values.

The relative site-specific loss of fat mass was compared using the ratio of trunk fat to leg fat. The ratio was higher in men at baseline (1.9 ± 0.068 vs. 1.3 ± 0.040, *P* < 0.01 for difference), but declined more rapidly in men over the course of the intervention, irrespective of diet (Figure 2).

## **Discussion**

The present study investigated sex differences in body compositional changes in response to a PRO weight loss diet compared to an isocaloric CARB weight loss diet in middle-aged adults. The main finding of this study was that diet and sex impacted changes in body composition independently and additively for the whole body, trunk and leg FM. No interactions of diet and sex were found on either whole body or regional body composition suggesting that males and females respond similarly to caloric restriction diets differing in protein content. To our knowledge, this is the first study to examine whether a higher protein diet can confer differences between men and women in degree or location of FM and LM reductions.

Some studies suggest that higher levels of protein in the diet may have a direct influence on degree of lean mass lost during energy restriction [9,10,14]. Because men generally have more LM, their absolute protein needs may be greater than in women; therefore, providing adequate protein during weight loss may be important in preserving LM. Contrary to our present results, Farnsworth et al., found that LM is preserved in women, but not in men on a higher protein diet [9]. The study by Farnsworth [9] was limited by a small number of men and a shorter duration (16 weeks total) than in the present study. Also, different protein levels between studies may partly explain the different findings. The higher protein diet in the Farnsworth study provided approximately 110 g protein/d for both men (~1.0 g kg^-1^ d^-1^) and women (~1.2 g kg^-1^ d^-1^) [9].

Conversely, protein intakes in the current study were slightly higher in men (PRO: 130 g d^-1^, 1.3 g kg ^-1^ d^-1^. CARB: 75 g d^-1^, 0.8 g kg^-1^ d^-1^) than in women (PRO: 100 g d^-1^ or 1.2 g kg^-1^ d^-1^. CARB: 64 g d^-1^, 0.7 g kg^-1^ d^-1^). One possible explanation for this discrepancy is that a threshold for LM maintenance was met in the current study, but not by the slightly lower protein intake relative to body weight reported by Farnsworth [9]. A threshold effect for maintaining LM during negative energy balance has not been characterized; however, a previous report in post-menopausal women demonstrated that for every additional 0.1 g kg ^-1^ d^-1^ of dietary protein intake, 0.62 kg LM was preserved during a 20-wk weight loss intervention [23]. The range of protein intakes was below the current RDA for protein intake [23]. Notably, a subsequent study demonstrates that protein intake of 1.5 g kg^-1^ d^-1^, which is above the current RDA, suppresses proteolysis thus inhibiting loss of lean mass [24,25].

Although men lost more total weight than women, when expressed relative to baseline body weight both sexes lost similar amounts of weight (~10 %). Importantly, we found that more of the total weight loss was derived from fat relative to LM in men (63 % and 77 % for CARB and PRO, respectively) than women (57 % and 67 %), and that more fat relative to lean was lost in PRO participants of both sexes. This is similar to previous findings [26], whereas other studies report no such effect of diet [9]. Although some studies show that sex does not influence the composition of weight loss from energy restriction [27,28], this finding is inconsistent [29,30]. In men, our results are similar to those reported in the literature, with the expectation that ~70 % of weight loss is comprised of FM during dieting alone [31]. Women in the PRO group also compared similarly to what was expected for FM loss, while the women in the CARB group lost less FM. However, we found no significant impact of the diets on sex differences in whole body weight, FM or LM loss.

Given the well-known association between central adiposity and CVD and metabolic complications [32-35], identifying changes in body fat distribution with weight loss is of particular interest. It is well established that women have greater levels of adiposity than men [1], and the distribution of fat differs, with men storing a greater proportion centrally and women in the gluteofemoral region [1]. However, with regard to the fat patterning, sex differences in changes in regional adipose depots through energy restriction are not well characterized. It appears that while both men and women lose FM from the abdominal area [7,36] and the femoral region [9], men may lose more abdominal fat [9,37] and women lose more femoral FM [7], even when matched on total weight and total fat loss [29]. Our data support the sex disparity in region of fat loss. Although both men and women reduced the relative fat of the whole body, trunk and leg, and no significant sex difference in the effect of the diets was found, these improvements are more pronounced in men, with the greatest change found in the PRO male group. Further assessment of the changes in the distribution of fat loss, indicated men experienced a reduction in the ratio of trunk fat to leg fat, suggesting that a greater degree of fat loss was derived from the central region.

Little evidence is available in the literature on potential underlying mechanisms to explain regional differences in body composition changes between men and women undergoing weight loss. However, one possible explanation for differences between men and women in their response to a higher protein weight loss diet could be related to a greater post-meal Diet Induced Thermogenesis (DIT) found in men compared to women [38,39]. Another explanation could be related to recent findings from an animal study, which showed that the protein content of a meal is the trigger for muscle protein synthesis, and this was shown to significantly increase energy expenditure, as measured by changes in adenosine 5’-triphosphate (ATP) and the signaling molecule adenosine monophosphate-activated protein kinase (AMPK) [40]. Importantly, the content of the branch chained amino acid leucine in a higher protein diet plays a key role in mechanisms that support the preservation of lean mass [41]. Other mechanisms, such as hormonal differences between sexes, may be responsible for the observed differences in body composition changes between men and women. Unfortunately, data from the present study are insufficient to examine underlying mechanisms.

Even though the sample size in the present study is larger than in most other studies, a diet by gender interaction may be only detectable with a larger baseline sample, which suggests that the effect of diet on sex differences in composition may be small. Men may benefit more from a greater protein intake during weight loss due to greater LM compared to women. A study designed to provide protein per kg of LM rather than in absolute terms or per kg body weight could elucidate this issue. Another consideration for our results is that the age range in the current study spans across menopausal status in women, potentially allowing hormonal status to influence the distribution of fat loss. Although our study was not designed to evaluate the impact of hormonal status or age per se on treatment effects, weight loss treatments that maximize FM loss while maintaining LM, especially in the legs are critical to combat the rising incidence of sarcopenic obesity. Indeed, statements in the literature regarding body composition and older adults indicate that this is a high research priority [11,42]. Lastly, more accurate measures of regional body composition, such as with computed tomography or magnetic resonance imaging, could elucidate differences in loss of FM from the abdominal vs. gluteofemoral regions between men and women and perhaps differences in changes in LM, especially in the lower body which is critical for physical function.

In summary, although we found no significant sex differences in the effects of diet on how much and where FM and LM are reduced during weight loss states, there is some evidence that protein intake levels may impact the amount of LM preserved during weight loss, perhaps regardless of sex. Clearly, further intervention studies, designed to assess interactive effects of sex and macronutrient content of the diet, are required to determine whether protein intake recommendations under energy restriction need to be adjusted to help maintain LM while losing FM, especially in populations such as older adults who are at higher risk for sarcopenia.

## **Authors’ contributions**

MM and RV participated in the study coordination and data collection, and drafted the manuscript. MT performed the statistical analysis and participated in drafting the manuscript. DL, PK-E and EE conceived of and designed the study, and participated in its coordination as well as helped draft the manuscript. All authors read and approved the final manuscript.

## Funding sources

Illinois Council on Food and Agricultural Research, National Cattlemen’s Beef Association, The Beef Board, Kraft Foods, National Science Foundation (PI: Layman).

## **Competing interests**

Dr Layman has participated in speaker bureaus for the National Cattlemen’s Beef Association and the National Dairy Council.
